# Family Physicians as System-Specific Specialists in Japan’s Aging Society

**DOI:** 10.7759/cureus.30811

**Published:** 2022-10-28

**Authors:** Ryuichi Ohta, Chiaki Sano

**Affiliations:** 1 Communiy Care, Unnan City Hospital, Unnan, Shimane, JPN; 2 Community Medicine Management, Shimane University Faculty of Medicine, Izumo, JPN

**Keywords:** japan, systems specialist, systems approach, role, definition, family medicine

## Abstract

Family medicine is a vital medical specialty in an aging society. The scope of each family doctor’s training and practice varies according to the context of their work, their roles, the organization and resources of the health systems in each country, and their ability to adapt to the healthcare needs of their country. As societal requirements change, so does the role of family medicine. In Japan, family physicians’ educational systems were officially established in 2017 as the nineteenth specialty, following discussions among family physicians, general internal medicine physicians, and hospitalists. Family physicians’ specialization in systems could facilitate access to appropriate health resources with proper timing while respecting the culture and context of each patient. Therefore, family physicians can be systems specialists on the same basis as organ and other specialists. Family physicians include people and families in their ecological systems. Family physicians should therefore specialize in healthcare systems.

## Editorial

Family medicine is a vital medical specialty in an aging society. Since the 1910s, family medicine has become a popular medical specialty worldwide, and family physicians have become the primary healthcare providers in communities [1]. The primary care provided by family physicians has improved healthcare quality regarding patient health and cost performance. To drive family medicine education, a definition of family medicine and competency-based education are essential to educating medical students in family medicine [1]. Each country determines the competencies of family physicians based on their contexts, and education in family medicine is based on family medicine competencies. Many definitions of family medicine are available in different settings worldwide, which may be affected by various factors.

Broadly, WONCA (the World Organization of Family Doctors) defines family physicians as practitioners who care for patients of all ages; ensure access to comprehensive primary and secondary services; manage infectious and chronic diseases; provide emergency, active, and long-term care; and coordinate individual clinical, community, and public health services. As McWhinney, the father of family medicine, stated, "Family physicians have in common the fact that they obtain fulfillment from personal relations more than from the technical aspects of treatment. Their commitment is more to a group of people than to a body of knowledge. Their experience gives them a distinctive perspective on illness, including its personal and social context [2]." Family physicians work based on these concepts and fit their abilities to their working situations and patients’ needs.

The scope of each family doctor’s training and practice varies according to the context of their work, their roles, the organization and resources of the health systems in each country, and the ability of those systems to adapt to the healthcare needs of their country [3]. In the United States and Europe, family physicians should honor the concept of equity and give patients equal access to equal services for identical needs while paying attention to the need to set medical priorities and show due responsibility for the appropriate use of resources [3]. This condition is best achieved in systems that offer controlled access to specialists, preferably secured via a "gate-keeping" system staffed by general practitioners who know their duties and limitations. Where gatekeeping roles do not exist, helping patients choose and obtain appropriate care from different parts of the system remains an essential function. Moreover, the definition implies that family physicians’ attention and efforts should focus on the entire healthcare system, from prevention through the detection of the first symptom to definitive treatment [4]. In Africa, holistic, longitudinal, comprehensive, family-oriented care and community orientation are respected based on the Delphi method [5,6]. Moreover, the contribution of family medicine to equity, quality, and primary healthcare within an African context should be encouraged, and the family physician's role as an educator should be promoted [6].

The role of family medicine is changing along with societal needs. Many family and general physicians have previously treated various medical conditions [2]. As medicine has advanced and specialization has increased, the overall number of specialists in specific organs has increased compared to the number of family physicians. Family physicians specialize in holistic approaches to patients and do not specialize in diseases [2]. Furthermore, with the increase in the number of aging societies, various medical issues such as multimorbidity, polypharmacy, and palliative care at home have increased in frequency [1]. Advancements in technology have driven medical professionals to specialize, and older patients now tend to be treated by multiple specialists. As information sharing among these professionals may not be adequate, fragmentation of care may complicate the treatment of older patients. Family physicians effectively manage these complex situations by respecting the psychosocial aspects of each patient’s care [1]. The process of the development and advancement of family medicine should not be confined solely to issues such as ethos, role traits, and gatekeepers. Developments in the academic field should be reflected in depth in these processes, and family physicians should perform reflective practices to promote contemplation of the future of family medicine [7].

In Japan, family physicians’ educational systems were officially established in 2017 as the nineteenth specialty, following discussions among family physicians, general internal medicine physicians, hospitalists, and primary care physicians [8]. "General medicine" is the nomenclature for this specialty. General medicine has six competencies: comprehensive care/integrated care, person-centered care, interprofessional collaboration, community orientation, professionalism, and system-based practice, which were developed taking WONCA competencies into consideration [9]. These competencies can be applied to various clinical settings for family physicians in Japan. However, the lack of specific educators for family medicine in Japan is an issue for family medicine education in general [10]. The training and development of such educators are vital for enhancing family medicine in Japan.

Furthermore, as in other countries, the relationship between family physicians and other medical specialists may not be smooth. This may be due to an insufficiently clear or complete understanding of family medicine among specialists and other professionals [11,12]. Based on various competencies from other countries, family physicians should be specialists in the whole person, community, and other system-related aspects of healthcare [3]. Family physicians’ specialization in person as a system can improve the comprehensive care of patients dealing with multimorbidity, polypharmacy, and polydoctorship. Family physicians’ specialization in health care as a system can facilitate access to appropriate health resources with proper timing while respecting the culture and context of each patient. Therefore, I assert that family physicians can be system-specific specialists like organs or other specialists.

Family medicine is the first gateway to primary healthcare in health systems and should organize patient health conditions. In the present Japanese medical conditions of free access, patients can freely go to various medical institutions. In particular, older patients tend to visit multiple organ-specific specialists because of multiple symptoms associated with multimorbidity, making the issue of polydoctor. Multiple prescriptions from each specialist can cause polypharmacy, which may impair their health conditions. Through the effective provision of family medicine, the present situation can be considered within the framework of a systems approach [1] whereby various medical specialists work in clinical settings. Family medicine can effectively care for various diseases in a person simultaneously [1]. Family physicians specializing in people as a system can manage multimorbidity and polydoctors causing polypharmacy and complications. To specialize in people as a system, family physicians should collaborate with various organ-specific specialists because less collaboration among the specialists may negatively influence healthcare systems. In the present situation, because of the short history of family medicine in Japan, family physicians may not be relied on by other organ-specific specialists [8,12]. Training in various clinical contexts and continuing high-quality care in family medicine can mitigate the gap between system- and organ-specific specialists, improving older patient care.

As a specialist in healthcare as a system, family physicians should collaborate with various healthcare professionals effectively as a leader of healthcare teams in their communities. The present healthcare system is dependent on the support provided by community healthcare professionals, and family physicians should respect their efforts and collaborate with them more effectively to promote current primary healthcare [1]. Family physicians should combine individuals and families in a holistic way and should facilitate the proper allocation of healthcare resources in communities, as all such resources are interconnected and their use and distribution have a systemic effect. Family physicians can specialize in the interactions of various healthcare factors and facilitate their interaction as a system, leading to better comprehensive care [13]. For effective learning of collaboration regarding older patient care, family medicine training should be performed in communities involving multiple healthcare professionals, older patients, and their families. Family medicine education in communities can drive students and residents to understand multiple professionals’ roles and reflect on their own roles as family physicians in communities [11,12,14].

Therefore, the development and advancement of family medicine should include citizens, community professionals, and various healthcare resources and acquire new perspectives in terms of developing family medicine system-specific specialists. The number of family physicians is also limited, and primary healthcare cannot be effectively performed. The motivation of medical students and residents to become family physicians in Japan may also be low because of the vagueness of the specialty of family medicine in advanced medical science [8,10]. To improve their motivation and the quality of family medicine, family medicine education should be driven, focusing on a system-specific specialist for people and healthcare systems.
